# CCAAT/Enhancer-Binding Protein Delta Regulates Glioblastoma Survival through Catalase-Mediated Hydrogen Peroxide Clearance

**DOI:** 10.1155/2022/4081380

**Published:** 2022-08-18

**Authors:** Hong-Yi Lin, Sher-Wei Lim, Tsung-I Hsu, Wen-Bin Yang, Chi-Chen Huang, Yu-Ting Tsai, Wen-Chang Chang, Chiung-Yuan Ko

**Affiliations:** ^1^Ph.D. Program for Neural Regenerative Medicine, College of Medical Science and Technology, Taipei Medical University and National Health Research Institutes, Zhunan, Taiwan; ^2^Graduate Institute of Medical Sciences, College of Medicine, Taipei Medical University, Taipei, Taiwan; ^3^Institute of Biomedical Sciences, National Sun Yat-sen University, Kaohsiung, Taiwan; ^4^Departments of Neurosurgery, Chi-Mei Medical Center, Tainan, Taiwan; ^5^Department of Nursing, Min-Hwei College of Health Care Management, Tainan, Taiwan; ^6^Ph.D. Program in Medical Neuroscience, College of Medical Science and Technology, Taipei Medical University and National Health Research Institutes, Taipei, Taiwan; ^7^Graduate Institute of Neural Regenerative Medicine, College of Medical Science and Technology, Taipei Medical University, Taipei, Taiwan; ^8^Neuroscience Research Center, Taipei Medical University, Taipei, Taiwan; ^9^TMU Research Center of Cancer Translational Medicine, Taipei, Taiwan

## Abstract

It has long been documented that cancer cells show increased and persistent oxidative stress due to increased reactive oxygen species (ROS), which is necessary for their increased proliferative rate. Due to the high levels of ROS, cancer cells also stimulate the antioxidant system, which includes the enzymes superoxide dismutase (SOD), catalase (CAT), and glutathione peroxidase (GPX), to eliminate ROS. However, overexpressed antioxidant enzymes often lead to drug resistance and therapeutic failure. Glioblastoma (GBM) is the most aggressive brain tumor and has the poorest prognosis. The transcription factor CCAAT/enhancer-binding protein delta (CEBPD) is highly expressed in GBM and correlates with drug resistance, prompting us to elucidate its role in GBM cell survival. In this study, we first demonstrated that loss of CEBPD significantly inhibited GBM cell viability and increased cell apoptosis. Furthermore, the expression of CAT was attenuated through promoter regulation following CEBPD knockdown, accelerating intracellular hydrogen peroxide (H_2_O_2_) accumulation. In addition, mitochondrial function was impaired in CEBPD knockdown cells. Together, we revealed the mechanism by which CEBPD-mediated CAT expression regulates H_2_O_2_ clearance for GBM cell survival.

## 1. Introduction

Glioblastoma (GBM) is the most malignant brain tumor, and its resistance to radiation and chemotherapy has been attributed to a variety of mechanisms, including radioresistance [1], glioma stem cells (GSCs) [2], enhanced DNA repair mechanisms [3], and altered antioxidant enzyme expression [4]. This resistance results in poor patient survival. A complex network of antioxidant enzymes prevents cellular damage by scavenging potentially harmful reactive oxygen and nitrogen oxide species (ROS/RNOS) that may damage cellular DNA, lipids, and proteins to maintain redox homeostasis. Under normal physiological conditions, ROS, such as superoxide, hydroxyl radicals, and hydrogen peroxide (H_2_O_2_), are produced mainly in mitochondria during cellular respiration and mediate the stimulation of various signaling pathways according to environmental conditions [5]. Increasing evidence supports a functional role for ROS in signaling cascades that promote proliferation, differentiation, and cell death [6–8]. Cellular redox imbalance between oxidants and antioxidants produces large amounts of ROS that are involved in the maintenance of genetic instability within tumor cells, including GBM [9].

Catalase (CAT) is a 240 kDa tetrameric enzyme localized predominantly in peroxisomes, where high concentrations of H_2_O_2_ are generated by numerous oxidases in virtually all aerobic organisms [10]. CAT protects against oxidative stress by preventing the accumulation of H_2_O_2_ and reducing it to water and oxygen. Meanwhile, elevated expression levels of CAT have been reported in cancer tissues compared to normal counterparts [11, 12], whereas other studies showed decreased levels of CAT [13, 14], indicating that cancer cells are frequently more sensitive to oxidative stress. In gliomas, catalase appears to be constitutively overexpressed compared with astrocytes [15]. Nevertheless, the molecular mechanism regulating the expression of CAT in GBM has not been fully elucidated.

CCAAT/enhancer-binding protein delta (CEBPD) is a transcription factor that plays important roles in inflammatory disease and cancer development [16, 17]. Our previous studies showed that CEBPD functions as a tumor suppressor by inducing cell growth arrest and apoptosis in some cancers [18, 19]. However, some studies suggest that CEBPD plays a prooncogenic role in regulating drug resistance and cell invasion [20, 21]. Recently, we demonstrated that CEBPD facilitates glioma stem cell formation by regulating stemness genes and elevates ATP-binding cassette subfamily A member 1 expression in temozolomide- (TMZ-) resistant GBM cells [22]. It has been reported that the expression of CEBPD reduced cisplatin-induced ROS and apoptosis in bladder urothelial carcinoma cells by inducing Cu/Zn-superoxide dismutase (SOD1) [20]. We also found that astrocytic CEBPD can increase extracellular ROS by directly regulating the *NCF1* and *NCF2* genes and provides an antioxidant effect for astrocytes resistant to intracellular ROS via activation of *SOD1* gene expression under inflammatory conditions [23]. These studies show that CEBPD is important in regulating redox balance. However, whether CEBPD regulates redox homeostasis for GBM development is unclarified.

In this study, we used a loss-of-function approach to demonstrate that CEBPD is essential for GBM cell survival. Increased caspase 3/7 expression and activity, decreased cell viability, H_2_O_2_ accumulation, and mitochondrial dysfunction were found in CEBPD knockdown GBM cells. According to a bioinformatic dataset analyzing relative gene expression in GBM cells, we found that CEBPD affects a subset of redox homeostasis-related genes. We further demonstrated that CEBPD regulates CAT expression through transcriptional regulation to protect against oxidative stress in GBM cell survival. Taken together, our results suggest that the CEBPD-CAT axis is a potential therapeutic target in GBM treatment.

## 2. Materials and Methods

### 2.1. Materials

TRIzol™ RNA extraction reagent, Lipofectamine® 2000 transfection reagent, Lipofectamine® RNAiMAX transfection reagent, Opti-MEM medium, Dulbecco's modified Eagle's medium (DMEM), fetal bovine serum (FBS), and antibody against GFAP (13-0300) were purchased from Thermo Fisher Scientific (Waltham, MA USA). The PrimeScript™ RT reagent kit was purchased from TaKaRa (Kusatsu, Shiga, Japan). SensiFAST™ SYBR was purchased from Bioline (Taunton, MA, USA). Antibodies against *α*-tubulin (T6199), *β*-actin (A5316), and HA-tag (SAB4300603) were purchased from Sigma (St. Louis, MO, USA). An antibody against CEBPD (SC-636) was purchased from Santa Cruz Biotechnology (Santa Cruz, CA, USA). An antibody against GAPDH (60004-1) was purchased from Proteintech Group, Inc. (Rosemont, IL, USA). Antibodies against cleaved caspase 3 (#9661) and catalase (#12980) were purchased from Cell Signaling Technology (Danvers, MA, USA). All oligonucleotides were synthesized by PURIGO Biotechnology (Taipei, Taiwan). HA-tagged CAT plasmid was purchased from Sino Biological Inc. (Beijing, China).

### 2.2. Cell Culture

The human GBM cell lines T98G and U373MG were purchased from the American Type Culture Collection (ATCC) and cultured in Dulbecco's modified Eagle medium (DMEM, Thermo Fisher Scientific) containing 10% fetal bovine serum (FBS, Thermo Fisher Scientific), 100 units/mL penicillin, and 100 *μ*g/mL streptomycin. We confirmed the authentication of all cell lines by short tandem repeat (STR) analyses of cell DNA alleles. The mycoplasma contamination test was examined by PCR analysis, and the result showed mycoplasma was not detected in all cells.

### 2.3. Western Blot Analysis

Cells were harvested and lysed with Pierce RIPA buffer (Thermo Fisher Scientific). Following lysis, the lysates were resolved on an SDS polyacrylamide gel and transferred to a polyvinylidene difluoride membrane by an electroblot apparatus. Membranes were incubated with primary antibodies overnight at 4°C and secondary antibodies at RT for 1 h. Proteins were detected by an enhanced chemiluminescence Western blot system from Pierce (Rockford, IL, USA) and visualized by an autoradiographic film.

### 2.4. Real-Time Quantitative Reverse Transcriptase PCR

The total RNA was harvested and extracted TRIzol™. The isolated RNA was subjected to reverse transcription with PrimeScript™ for cDNA synthesis. Real-time PCR was conducted using iTaq Universal SYBR Green Supermix. PCR was conducted using StepOne Plus™ real-time PCR systems (ABI) with the following pairs of specific primers: human GAPDH forward 5′-CCACCCAGAAGACTGTGGAT-3′ and reverse 5′-TTCAGCTCAGGGATGACCTT-3′, human CEBPD forward 5′-GCCATGTACGACGACGAGAG-3′ and reverse 5′-TGTGATTGCTGTTGAAGAGGTC-3′, and human CAT forward 5′-GTGCGGAGATTCAACACTGCCA-3′ and reverse 5′-CGGCAATGTTCTCACACAGACG-3′. All reactions were performed in duplicate with “no reverse transcriptase” as the control, and all data are expressed as the mean ± SEM of at least three independent biological replicates. The relative expression levels were measured using the relative quantitation (RQ) *ΔΔ*Ct method and normalized to the housekeeping gene GAPDH.

### 2.5. Establishment of Stable Knockdown Clones

Virus was produced from Phoenix cells by cotransfection of the various small hairpin RNA expression vectors in combination with pMD2.G and psPAX2. After determining the viral infection efficiency, 10 multiplicities of infection of lentivirus containing shLuciferase (shLuc, for knockdown control) or shCEBPD (shB7 and shC7) were used to infect U373MG or T98G cells for 96 h. Cells were further diluted into 96-well culture plates (10 cells/well) and incubated with G418 (400 *μ*g/ml) selection medium. Cells were feed every 4 days with selection medium. Resistant cell clones were obtained about 30 days and maintained in G418-containing (100 *μ*g/ml) culture medium. The expression of CEBPD was further confirmed by Western blot analysis. In all lentiviral experiments, the medium containing uninfected viruses was removed before further assays were conducted. The small hairpin RNA sequences in lentiviral expression vectors were as follows: shLuciferase (shLuc): 5′-CCGGCTTCGAAATGTCCGTTCGGTTCTCGAGAACCGAACGGACATTTCGAAGTTTTTG-3′, shCEBPD (shB7): 5′-CCGGGCCGACCTCTTCAACAGCAATCTCGAGATTGCTGTTGAAGAGGTCGGCTTTTT-3′, and shCEBPD (shC7): 5′-CCGGGCTGTCGGCTGAGAACGAGAACTCGAGTTCTCGTTCTCAGCCGACAGCTTTTT-3′. The lentiviral knockdown expression vectors were obtained from the National RNAi Core Facility located at the Genomic Research Center of Institute of Molecular Biology, Academia Sinica (Taiwan). For the maintenance of control or CEBPD knockdown cells, cells were seeded at similar numbers and passaged at 90% confluence (control cells: 3 days and CEBPD knockdown cells: 5 days). The CEBPD knockdown cells were passaged less frequently.

### 2.6. Plasmid Transfection and Reporter Assay

Cells were replated 24 h before transfection at an optimal density in 2 ml of fresh culture medium in a 6-well plastic dish. They were then transfected with plasmids by Lipofectamine® 2000 transfection reagent according to the manufacturer's instructions. The total amount of DNA for each experiment was matched with the empty vector. The Opti-MEM media were changed to culture medium after 6 h, incubated for 15 h, and harvested for further analysis. The serial fragments and mutants of 5′ promoter region on *CAT* gene were synthesized by MDBio, Inc. (Taipei, Taiwan) and cloned into pGL-3 basic vector. After transfection, the luciferase activities in cell lysates were measured following the manufacturer's instructions for the luciferase assay.

### 2.7. Small Interfering RNA (siRNA) Assay

The sequence of CEBPD knockdown si2895 was 5′-UUCUCUCGCAGUUUAGUGGTG-3′, si2896 was 5′-AUUGCUGUUGAAGAGGUCGGC-3′ (Thermo Fisher Scientific), and a negative control siRNA sequence (Dharmacon, D-001810-01-05) was not found in the human genome databases. Cells were transfected separately with CEBPD siRNA or negative control siRNA by Lipofectamine® RNAiMAX transfection reagent according to the manufacturer's instructions. After 48 h, cells were harvested for further analysis.

### 2.8. Microarray Analysis and Bioinformatic Analysis for Gene Expression

Total RNAs were isolated using the TRIzol RNA extraction reagent. Samples were validated with SurePrint G3 Human whole genome microarray 8 × 60 K [(60000 probes, including 25045 genes (Agilent, G4450A)], following the manufacturers' protocols. All processes were performed by Welgene Biotech Company (Taipei, Taiwan). Good quality signals were obtained by filtering for scores of *p* value < 0.05 in all replicates, *M* value of >6 in all signals, and more than 1.5-fold change. Finally, the function of candidate genes was assigned by Ingenuity Pathway Analysis (IPA) (Ingenuity Systems Inc., Redwood City, CA, USA). Heatmaps were prepared based on the level of expression using ToppCluster (https://toppcluster.cchmc.org/). The mRNA expression was analyzed using the GEPIA2 website (http://gepia2.cancer-pku.cn/#index) with TCGA_GBM dataset.

### 2.9. Gene Expression Omnibus (GEO) Database

The GEO databases used in this study are the Lee dataset (GSE4536) [24], Sun dataset (GSE4290) [25], Murat dataset (GSE7696) [26], and Shai dataset [27]. These databases were used to assess gene expression levels in normal and glioma tissues.

### 2.10. Caspase 3/7 Activity Assay

The conditioned media were prepared from different cells, and then, an equivalent amount of Caspase-Glo® 3/7 reagent (Promega, WI, USA) was added to the 96-well plates. The samples were mixed on a shaker at room temperature for 30 min, and the luciferase activity was measured with a luminometer.

### 2.11. Catalase Activity Assay

Cells (10^6^) were lysed with assay buffer, and the supernatant was used for catalase activity with a catalase activity colorimetric/fluorometric assay kit (K773, BioVision, Inc., CA, USA) according to the manufacturer's instructions.

### 2.12. Cell Viability Assay

The CCK-8 assay was conducted using 96-well plates. Briefly, the cells were seeded into the 96-well plates at a density of 1000 cells per well. Three replicate wells were set up for each sample. Cell viability was examined daily for 4 d, consecutively, after cell seeding. On each day, 10 *μ*l of the CCK-8 solution was added to each well of cells. After 1 h of incubation, the absorbance of each well was measured using a microplate reader, after which the results were statistically analyzed.

### 2.13. Colony Formation Assay

Cells were plated on 6 cm culture dishes (1 × 10^3^ cells of U373MG or T98G) for 7 days. Cell colonies were stained with 0.05% crystal violet in 50% ethanol, and the colonies were photographed and analyzed with the ImageJ software.

### 2.14. Chromatin Immunoprecipitation (ChIP) Assay

In brief, cells were treated with 1% formaldehyde for 10 min, and the nuclear proteins were extracted. The cross-linked chromatin was then prepared and sonicated to an average length between 200 bp and 1000 bp. The DNA fragments were immunoprecipitated with specific antibodies recognizing CEBPD (sc-636x, Santa Cruz, CA, USA) or control rabbit immunoglobulin G (IgG) (sc-2027, Santa Cruz, CA, USA) at 4°C for 16 h. After reversal of the crosslinking between proteins and genomic DNA, the precipitated DNA was amplified by PCR with primers related to the specific regions on the genomic loci of target genes. The primers included -477 forward, 5′-GCTGAGAAAGCATAGCTATG-3′ and -252 reverse, 5′-AGGAGGGTGCGGAAAGGAAG-3′ and -188 forward, 5′-CAGCCAATCAGAAGGCAGTC-3′ and -5 reverse, 5′-TGCGGTTTGCTGTGCAGAAC-3′.

### 2.15. XFe24 Seahorse Mitochondrial Respiration Mito Stress Test

Cells were seeded into XFe24 cell culture microplates (1 × 10^4^ cells/well) and incubated for one day. Meanwhile, a sensor cartridge (detecting probes, Agilent Technologies) in Seahorse XF Calibrant at 37°C was hydrated in a non-CO_2_ incubator overnight for the following experiments. On the assay day, the cell culture medium was replaced with assay medium (DMEM without sodium bicarbonate, supplemented with 2% FBS and penicillin/streptomycin, pH 7.4) and incubated for 1 h. Cells were then incubated at 37°C in a non-CO_2_ incubator for experiments. Oligomycin (10 *μ*M), FCCP (2 *μ*M), and rotenone/antimycin A (5 *μ*M) were prepared and placed into the sensor cartridge for the injection in the running procedure. The procedure of the assay was performed according to the guidelines for the XFe24 Seahorse Mitochondrial Respiration Mito Stress Test (Agilent Technologies) [28].

### 2.16. Estimation of H_2_O_2_ Level

Cells were seeded on a 96-well plate at a density of 5,000 cells per well. H_2_O_2_ levels were detected by using an ROS-Glo™ H_2_O_2_ assay kit (G8820, Promega) according to the manufacturer's instructions. Samples were incubated with H_2_O_2_ substrate solution for 6 h, followed by the addition of ROS-Glo™ detection solution and incubation for 20 min. The luminescence units (RLU) were determined by a GloMax® Discover Microplate Reader (GM3000, Promega).

### 2.17. Xenograft Mouse Model

Male NOD. CB17-Prkdcscid/J mice (8 weeks old, BioLASCO Taiwan Co Ltd, Taipei, Taiwan) were housed at the animal facility of Taipei Medical University (TMU). All animal experiments were conducted in accordance with procedures outlined in the Guide for the Care and Use of Laboratory Animals and under the supervision of the Institutional Animal Care and Use Committee of TMU. A total of 2 × 10^6^ T98G cells with 50% Matrigel were injected subcutaneously into the right flank of mice. The body weight of each mouse and their corresponding tumor size were measured to observe how tumor progression impacted the health status of each mouse (Supplementary figure 1). Tumor diameters were measured at regular intervals with a caliper, and the tumor volume in mm^3^ was calculated weekly by the formula provided by the National Cancer Institute: tumor volume = 3.14/6 × length × width^2^.

### 2.18. Immunofluorescence (IF) Staining

The histological sections cut were sent for IF staining. The antigen retrieval program was incubated in boiling citrate buffer (pH 6) for 12 minutes. Blocking buffer (TA00C2, BioTnA, Kaohsiung, Taiwan) was used for 30 minutes. Tissue sections were stained with primary antibody for 16 hours, followed by fluorescence-conjugated secondary antibody. The TUNEL staining was conducted with TUNEL apoptosis assay kit (BioTnA, TAAP01F, Kaohsiung, Taiwan) according to the manufacturer's instructions.

### 2.19. Statistical Analysis

Results are shown as the mean ± SEM. All statistical analyses were conducted using the GraphPad Prism software. Student's *t*-test and one-way or two-way analysis of variance (ANOVA) followed by Tukey's multiple comparison test were used. The correlation analysis was determined by the Pearson correlation test. All experiments were repeated in triplicate. Statistically significant differences are indicated by ^∗∗∗^*p* < 0.001, ^∗∗^*p* < 0.01, and ^∗^*p* < 0.05.

## 3. Results

### 3.1. Downregulation of CEBPD Reduces Cell Viability in GBM Cells

**Figure 1:**
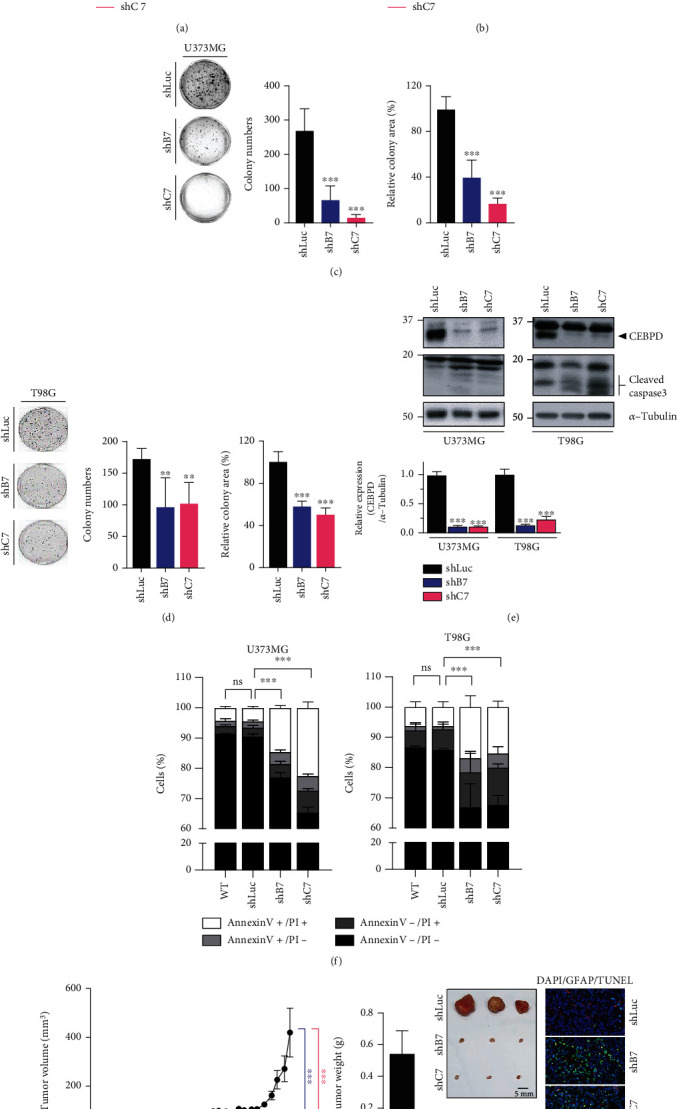
Loss of CEBPD attenuates cell viability and induces cell apoptosis in GBM. (a, b) Knockdown of CEBPD reduces GBM cell viability. Cells from U373MG or T98G stable clones were subjected to CCK-8 proliferation assays. (c, d) Stable knockdown clones of (c) U373MG or (d) T98G cells were subjected to colony formation assays and grown for 7 days. The quantitative results of colony numbers and size are shown in the middle and right panels. (e) Attenuated CEBPD increases cleaved caspase 3 expression in GBM cells. Western blot analyses were conducted with the indicated antibodies using protein lysates from U373MG or T98G stable clones. Expression of *α*-tubulin served as the internal control. Lower panel shows the quantification of CEBPD protein expression. (f) Cells were harvested from U373MG or T98G stable clones and stained with Annexin V and Propidium Iodide (PI) for flow cytometry analysis. (g) Cells (2 × 10^6^) from stable T98G clones were injected subcutaneously into NOD-SCID mice. The mouse brain was paraffin embedded and subjected to histological analysis (right panel). Brain slides were stained by GFAP antibody and TUNEL apoptosis assay and photographed by microscope. Bars represent the means ± SEM from three independent experiments. Differences among groups were determined with one-way or two-way ANOVA followed by Tukey's multiple comparison test. ^∗∗∗^*p* < 0.001 and ^∗∗^*p* < 0.01. ns: no significant; shLuc: shRNA for luciferase; shB7, shC7: shRNAs for CEBPD.

Our previous study showed that CEBPD is expressed at high levels in GBM patients, correlates with poor survival probability, and contributes to TMZ resistance [22]. To further clarify the functional role of CEBPD in GBM development, we generated CEBPD stable knockdown clones of U373MG and T98G cells. Knockdown of CEBPD significantly reduced the GBM cell viability both in stable clones or transiently siRNA-transfected cells (Figures 1(a) and 1(b) and Supplementary figure 2A). Moreover, the numbers and area of colonies in both cell lines were attenuated in knockdown clones compared to control (Figures 1(c) and 1(d)). These results indicate that CEBPD is vital for GBM cell survival. In addition, downregulation of CEBPD also increased cleaved caspase 3 levels (Figure 1(e)) and elevated caspase 3/7 activity (Supplementary figure 2B) in U373MG and T98G cells. To further clarify that the elevated caspase 3 expression is correlated to cell death, we costained the cells with Annexin V and Propidium Iodide (PI). We found that the ratio of early apoptosis cells (Annexin V+, PI-) and late apoptosis cells (Annexin V+, PI+) were both increased in CEBPD knockdown cells compared to knockdown control (Figure 1(f) and Supplementary figure 2C). We also analyzed the cell cycle status of control and CEBPD knockdown cells and found that sub-G1 populations were increased in CEBPD knockdown cells compared to control cells (Supplementary figure 3). Furthermore, we performed an *in vivo* study using a mouse xenograft model of CEBPD stable knockdown clones of T98G cells. Compared to the knockdown control, xenografts bearing CEBPD knockdown exhibited significantly inhibited tumor growth and elevated TUNEL staining (Figure 1(g)). Taken together, these data suggest that CEBPD plays important roles in GBM survival. Our findings are also consistent with previous study that CEBPD blocking peptide impairs cell growth/survival and induces cell apoptosis in GBM cells [29].

### 3.2. CAT Is Expressed at High Levels in GBM Patients and Positively Correlates with CEBPD Expression

**Figure 2:**
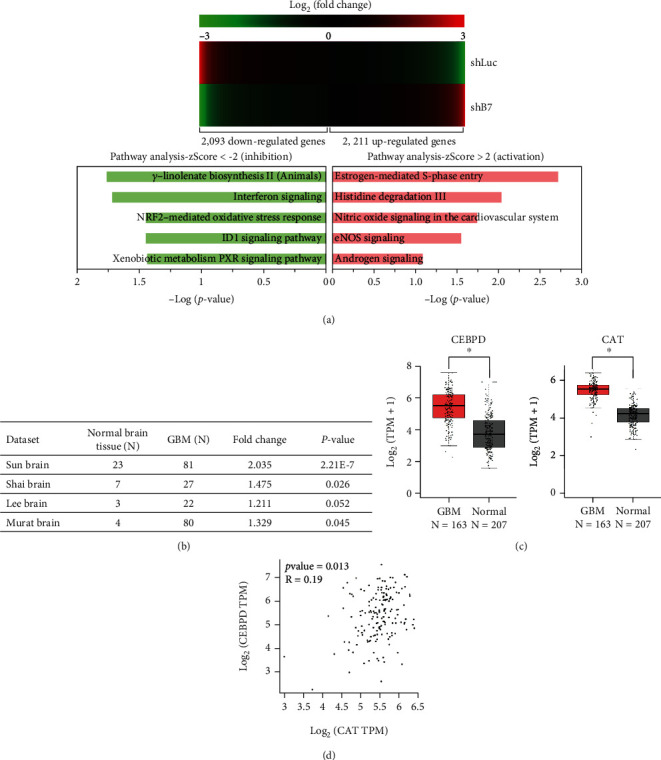
Relative CAT expression is higher and associated with CEBPD expression in GBM tissues. (a) Cells from U373MG stable clones were harvested and subjected to microarray analysis. Clustering of microarray data from RNA of cells as shown identified significant gene expression clusters resulting from CEBPD downregulation (upper panel). The IPA software program was applied on 4304 potential CEBPD-regulated genes to identify top 5 scoring canonical activation or inhibition pathways (lower panel). (b) Analyses of *CAT* mRNA expression from GEO databases in GBM and normal brain tissues. (c) The mRNA expression levels of *CEBPD* and *CAT* are higher in human GBM tissues according to TCGA_GBM database. (d) The mRNA expression of *CAT* positively correlates with *CEBPD* in GBM tissues according to TCGA_GBM database. The summary data are presented as the mean ± SEM; Student's *t*-test; ^∗^*p* < 0.05. shLuc: shRNA for luciferase; shB7: shRNA for CEBPD.

Cancer cells often acquire the ability to mitigate programmed cell death pathways and recalibrate the redox balance to survive. Herein, we attempted to elucidate whether CEBPD is involved in redox homeostasis for the survival of GBM cells. By analyzing the microarry-based transcriptome, 2211 genes were upregulated and 2093 genes were downregulated in CEBPD knockdown cells compared to luciferase knockdown control (Figure 2(a), upper panel). We also listed the top five upregulated and downregulated cellular pathways, respectively, using IPA analysis (Figure 2(a), lower panel). Redox homeostasis-related, including glutathione peroxidase (GPX) 3, 4, 6, 7, and 8, nuclear factor erythroid-derived 2-like 2 (NFE2L2, also known as nuclear factor erythroid 2-related factor 2, NRF2), thioredoxin reductases (TXNRD) 1 and 3, and CAT genes are downregulated in CEBPD stable knockdown clone (Table 1). GBM GEO datasets showed that *CAT* messenger RNA (mRNA) levels were higher in GBM tissues than in normal brain tissues (Figure 2(b)). Our previous study also shows the higher *CEBPD* mRNA levels in GBM tissues than in normal brain tissues in these GEO datasets [22]. Using TCGA_GBM database, the mRNA levels of *CEBPD* and *CAT* were significantly higher in GBM than in normal tissues and showed that the expression level of *CAT* correlated with the expression level of *CEBPD* in these GBM samples (Figures 2(c) and 2(d)).

### 3.3. The *CAT* Gene Is a Downstream Target of CEBPD

**Figure 3:**
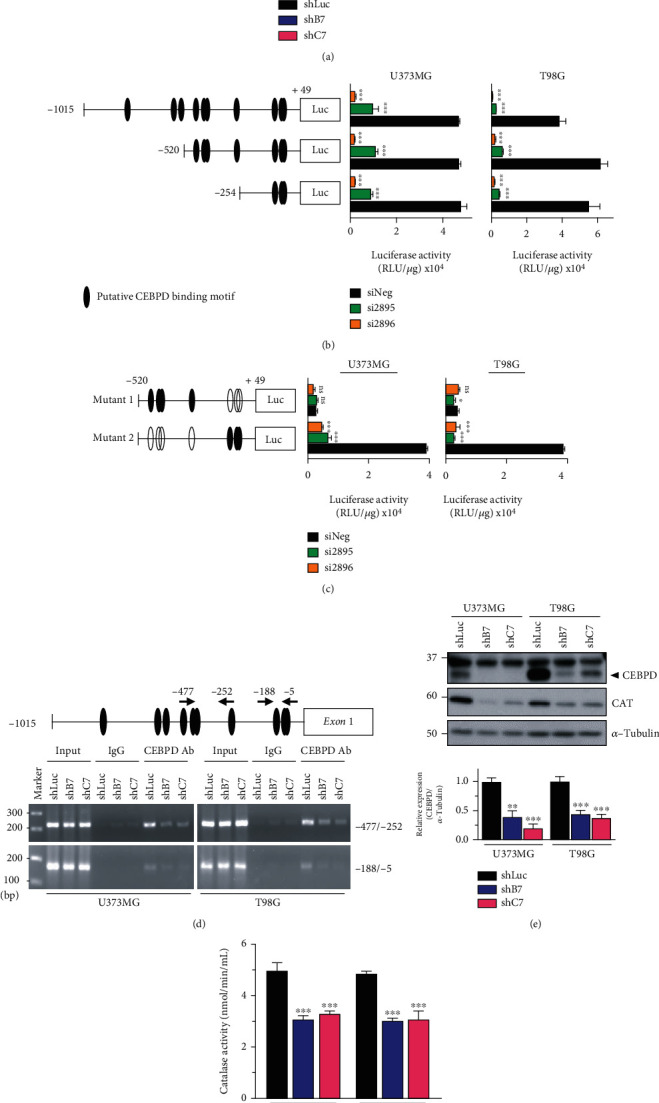
CEBPD regulates CAT expression. (a) Total RNA from U373MG or T98G stable clones was harvested and examined by RT-qPCR to detect *CAT* expression levels. (b) Left panel shows a schematic representation of the various *CAT*-based reporter constructs used in this study. Cells were cotransfected with the indicated *CAT* reporter constructs and siRNAs. After 72 h, cells were lysed for luciferase assay. (c) Left panel shows a schematic representation of the *CAT*-based reporter mutation constructs. Cells were cotransfected with the indicated *CAT* reporter mutation constructs and siRNAs. After 72 h, cells were lysed for luciferase assay. (d) CEBPD binds to the *CAT* promoter. Sheared formaldehyde cross-linked chromatin from U373MG or T98G stable clones was immunoprecipitated with the indicated antibodies and processed for PCR amplification. As a positive control, PCR amplification was also performed with input chromatin that was collected before the IP step. The chromatin was isolated from stable clones. An IP step was performed with IgG or CEBPD antibody. The “-477/-252” and “-188/-5” indicate the PCR products after amplification with specific primers using purified templates from the specific antibody-IP step. (e) Western blot analyses were conducted with the indicated antibodies using protein lysates from U373MG or T98G stable clones. Expression of *α*-tubulin served as the internal control. Lower panel shows the quantification of CAT protein expression. (f) Cells were harvested from U373MG or T98G stable clones and subjected to catalase activity analysis. Bars represent the means ± SEMs from three independent experiments. Differences among groups were determined with one-way ANOVA followed by Tukey's multiple comparison test. ^∗∗∗^*p* < 0.001, ^∗∗^*p* < 0.01, and ^∗^*p* < 0.05. ns: no significant; shLuc: shRNA for luciferase; shB7, shC7: shRNAs for CEBPD; siNeg: siRNA for negative control; si2895, si2896: siRNAs for CEBPD.

To further confirm that CAT is regulated by CEBPD, we analyzed the CAT gene and protein expression levels. The results showed decreased mRNA and protein levels of CAT in CEBPD knockdown cells (Figure 3(a) and Supplementary figure 4A). According to the prediction website for transcription factor binding (http://alggen.lsi.upc.es/cgi-bin/promo_v3/promo/promoinit.cgi?dirDB=TF_8.3), several CEBPD binding sites were identified in the *CAT* promoter regions (Figure 3(b), left panel). The promoter activity of *CAT* was downregulated in CEBPD knockdown cells (Figure 3(b) and Supplementary figure 4B, right panel). Moreover, to further clarify CEBPD responsive region, we used serial deletion *CAT* promoter constructs and found that the main CEBPD responsive region was during -254 to +49 of *CAT* promoter. We also mutated the proximal (mutant 1) and distal (mutant 2) CEBPD putative sites of *CAT* promoter and found that mutant 1 reporter showed no difference between CEBPD knockdown and control groups (Figure 3(c)). Moreover, the basal reporter activity of mutant 1 was greatly decreased compared to -520/+49 or -520/+49 mutant 2 reporter. We next conducted an *in vivo* DNA binding assay to assess the direct binding of CEBPD to the promoter of the *CAT* gene. The PCR results of the ChIP assay showed that CEBPD was directly bound the promoter of the *CAT* gene (Figure 3(d)). We also overexpressed CEBPD in CEBPD knockdown cells and found that attenuated *CAT* reporter activity could be elevated in CEBPD knockdown cell (Supplementary figure 4C). These results suggest that CEBPD regulates CAT expression through promoter regulation. Furthermore, lower CAT expression also correlated with lower total CAT activity in CEBPD stable knockdown clones (Figures 3(e) and 3(f) and Supplementary figure 4D).

### 3.4. CEBPD Regulates H_2_O_2_ Metabolism in GBM Cells

**Figure 4:**
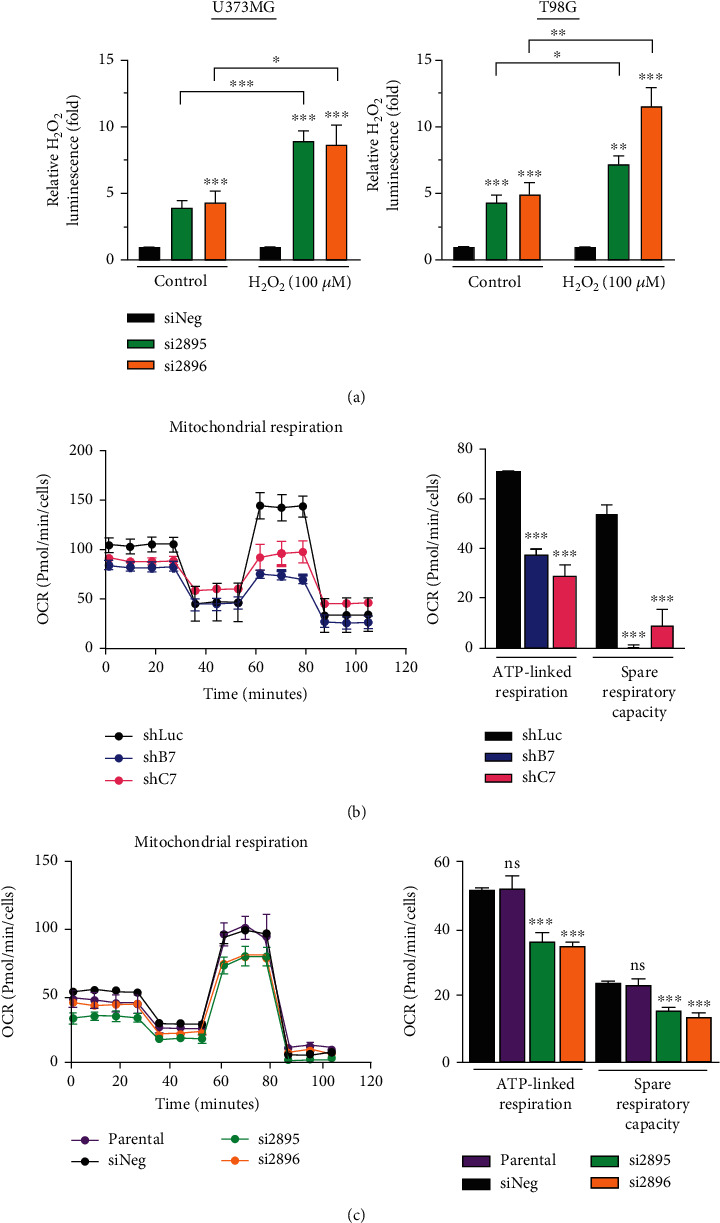
CEBPD knockdown elevates H_2_O_2_ accumulation and impairs mitochondrial function. (a) U373MG or T98G cells were transiently transfected with control siRNA or CEBPD siRNA. After 48 h, cells were treated with or without H_2_O_2_ for 6 h and then harvested for the measurement of H₂O₂ levels. (b, c) The mitochondrial activities of (b) CEBPD stable knockdown clone T98G or (c) CEBPD transient knockdown U373MG cells are shown as the oxygen consumption rate (OCR) determined by the Seahorse XF Mito stress test. Bars represent the means ± SEMs from three independent experiments. Differences among groups were determined with one-way or two-way ANOVA followed by Tukey's multiple comparison test. ^∗∗∗^*p* < 0.001, ^∗∗^*p* < 0.01, and ^∗^*p* < 0.05. ns: no significant; shLuc: shRNA for luciferase; shB7, shC7: shRNAs for CEBPD; siNeg: siRNA for negative control; si2895, si2896: siRNAs for CEBPD.

**Figure 5:**
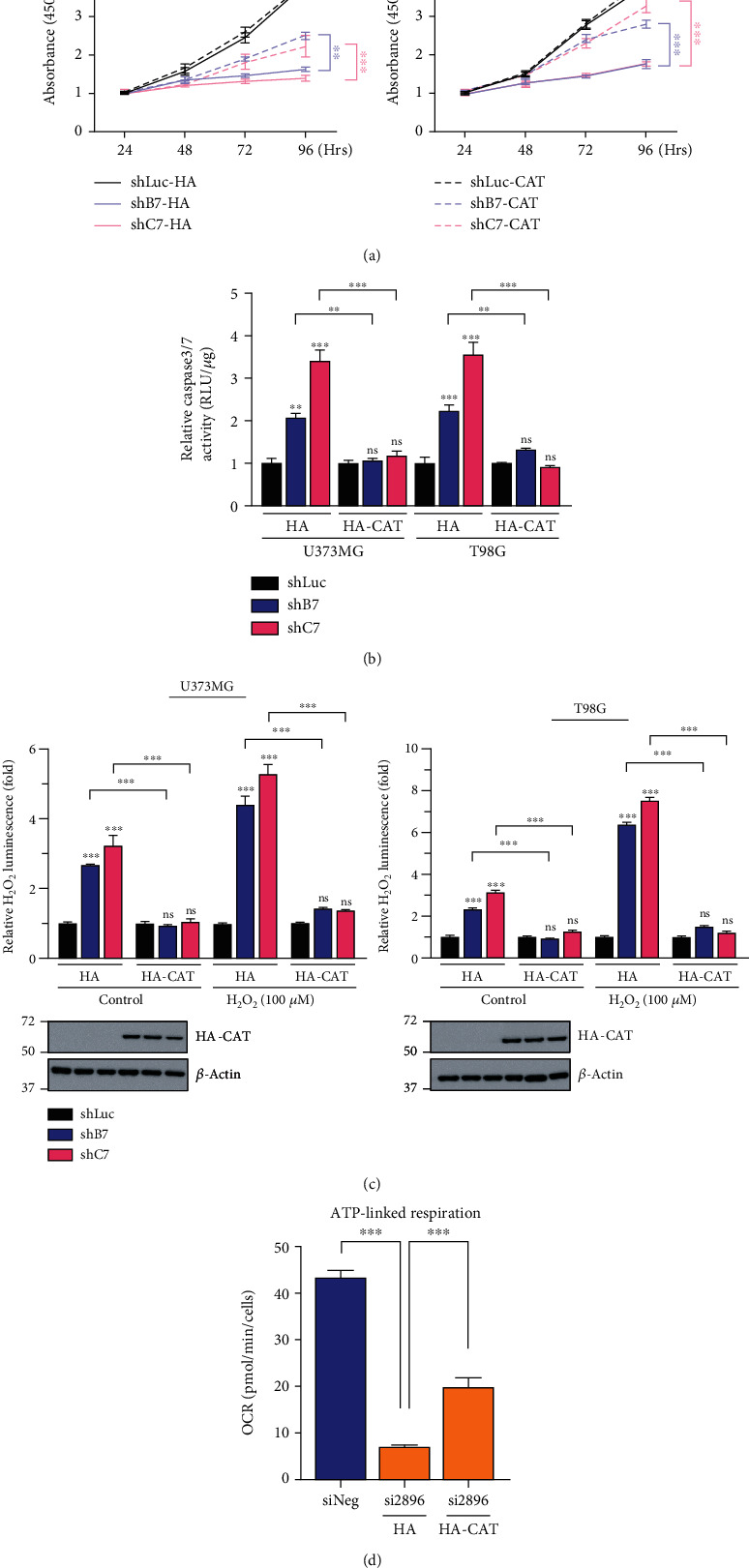
Overexpression of CAT decreases caspase 3/7 activity and ameliorates cell viability and mitochondrial function in CEBPD knockdown GBM cells. (a) The U373MG or T98G stable clones were transiently transfected with HA or HA-CAT for 24 h and then subjected to CCK-8 viability assay. (b) Stable knockdown clones of U373MG or T98G cells were transfected with HA or HA-CAT plasmids. After 24 h, cells were harvested and the caspase 3/7 activity was determined by Caspase-Glo® 3/7 reagent. (c) Stable knockdown clones of U373MG or T98G cells were transfected with HA or HA-CAT plasmids. After 24 h, cells were treated with or without H_2_O_2_ for 6 h and then harvested for the measurement of H₂O₂ levels. (d) The T98G cells were transiently cotransfected with control siRNA or CEBPD siRNA and HA or HA-CAT. After 72 h, cells were harvested and subjected to Seahorse XF Mito stress test to determine ATP-linked respiration. Bars represent the means ± SEMs from three independent experiments. Differences among groups were determined with one-way or two-way ANOVA followed by Tukey's multiple comparison test. ^∗∗∗^*p* < 0.001 and ^∗∗^*p* < 0.01. ns: no significant; shLuc: shRNA for luciferase; shB7, shC7: shRNAs for CEBPD; siNeg: siRNA for negative control; si2896: siRNA for CEBPD; HA: hemagglutinin; HA-CAT: HA-tagged catalase.

As an antioxidant enzyme, CAT catalyzes the conversion of H_2_O_2_ to water and oxygen. To clarify whether CEBPD affects CAT-mediated H_2_O_2_ metabolism, the levels of H_2_O_2_ were examined in CEBPD knockdown GBM cells. Following CEBPD knockdown, the levels of H_2_O_2_ were significantly increased compared to the knockdown control in both U373MG and T98G cells (Figure 4(a), control groups). Moreover, higher levels of H_2_O_2_ accumulated when cells were pretreated with H_2_O_2_ in the CEBPD knockdown groups (Figure 4(a), H_2_O_2_ groups). In addition, we found that CEBPD knockdown significantly attenuated mitochondrial respiration (Figures 4(b) and 4(c)). Ectopic expression of CAT rescued CEBPD knockdown-mediated attenuation of cell viability (Figure 5(a)). Furthermore, the caspase 3/7 activity and accumulation of H_2_O_2_ were significantly eliminated following CAT overexpression in CEBPD knockdown cells (Figures 5(b) and 5(c), HA-CAT groups compared to HA groups). The ATP-linked respiration was also partially restored with CAT overexpression in CEBPD knockdown cells (Figure 5(d)). These data suggest that CAT mediates CEBPD regulated cell survival and H_2_O_2_ metabolism in GBM cells.

## 4. Discussion

ROS paradoxically promotes cancer progression and induces detrimental cytotoxic effects. Within the CNS, astrocytes and neurons have antioxidant systems that protect these cells from oxidant damage; the mRNA expression of SOD and CAT enzymes is high in astrocytes. These differences in the expression of antioxidant enzymes make astrocytes particularly sensitive to damage induced by ROS, leading to genetic instability when the redox balance is lost. It is generally accepted that the cellular maintenance of redox homeostasis is controlled by a complex network of antioxidant enzymes (i.e., SOD and glutathione peroxidases) whose expression is under precise regulation by NRF2 [30]. CAT expression has been correlated with glioma resistance to the chemotherapeutic agent carmustine, a DNA alkylating agent [31]. It has been reported that intracellular ROS and extracellular H_2_O_2_ are increased and sensitivity to radiation, and H_2_O_2_ is increased in CAT knockdown glioma cells [32]. Nevertheless, the molecular mechanisms regulating the expression of CAT have not been totally elucidated. Previous study showed that CEBPB, NF-Y and Sp1, play an essential role in the positive regulation of CAT expression [33, 34]. In the current study, we provided evidence showing that CEBPD regulates CAT expression through promoter regulation to eliminate intracellular H_2_O_2_ for GBM survival. However, whether the CEBPD-CAT pathway protects GBM against TMZ-induced stress needs to be further elucidated. According to our previous study, CEBPD is downregulated in hepatocellular carcinoma (HCC) and cervical cancer, serving as a tumor suppressor [35]. It has been reported that CAT is also decreased in HCC but increased in cervical cancer. However, whether the CEBPD-CAT regulation axis also exists in HCC needs to be further clarified. On the other hand, Cebpd-deficiency promotes radiation-induced deficits in short-term memory and spatial learning in aged mice that may be due to an impaired ability to detoxify IR-induced oxidative stress mediated by decreased CAT [36]. These results demonstrated that CEBPD may regulate CAT both in tumor and normal cells.

As a transcription factor, CEBPD can be activated by inflammatory cytokines and anticancer drug treatments. Previously, we showed that CEBPD participates in the upregulation of the GSC stemness factors SOX2, OCT4, NANOG, and ABCA1 to contribute to TMZ resistance in GBM. In this study, we further showed that loss of CEBPD enhanced H_2_O_2_ accumulation, leading to cell apoptosis. An *in vivo* xenograft study also confirmed the decreased tumor growth by CEBPD inhibition. These findings indicate that CEBPD plays important roles in GBM development and drug resistance. As shown in Figure 1, inhibition of CEBPD attenuated viability and promoted apoptosis in GBM cells. These results are consistent with our previous study showing that overexpression of CEBPD upregulated antiapoptotic B-cell lymphoma 2 (BCL2) and the proliferation regulator c-MYC and downregulated proapoptotic BCL2-associated X protein (BAX) expression in glioma cells [37]. On the other hand, CEBPD may also regulate the expression of GPXs, NRF2, and TXNRDs to protect against oxidative stress in GBM. The detailed mechanism of how CEBPD comprehensively regulates redox balance in GBM development should be further verified.

In this study, we used two different target sequences to establish CEBPD knockdown stable clones. Although these knockdown stable clones still maintain low CEBPD expression for cell survival, there may be some uncharacterized adaptation or compensate effects from other CEBP family member for these survival clones. To eliminate this possibility, we used transient knockdown approach with siRNAs to clarify CEBPD function in GBM. Many results from transient CEBPD knockdown cells were consistent with stable clones, showing that CEBPD plays important role in GBM survival.

Many cellular signaling pathways, including those that drive cell division, interact tightly with the mechanisms that regulate mitochondrial function, including mitochondrial fission and fusion, mitochondrial biogenesis, mitochondrial activity, and mitochondrial apoptosis (intrinsic pathway). In addition to archetypal cell cycle regulators, key transcription factors that also play roles in proliferation and cell cycle arrest are also essential players in the regulation of mitochondrial function [38]. In this study, we found that the S and G2/M populations of CEBPD knockdown cells were similar to the control cells. However, the G0/G1 population was decreased and sub-G1 population was increased in CEBPD knockdown cells. These results showed that the impaired mitochondrial function in CEBPD knockdown cells is not due to less cell dividing or arrest.

## Figures and Tables

**Table 1:** CEBPD-regulated redox homeostasis-related genes.

Gene symbol	Entrez Gene ID	log_2_ratio (shB7/shLuc)	SNR^#^
NFE2L2	4780	-2.09	0.18
GPX6	257202	-1.95	0.13
CAT	**847**	**-0.93**	**39.8**
GPX7	2882	-0.35	0.69
GPX3	2878	-0.23	0.05
GPX8	493869	-0.23	16.6
TXNRD3	114112	-0.20	2.03
GSR	2936	-0.20	1.63
TXNRD1	7296	-0.16	2.45
GPX4	2879	-0.13	46.3
SOD1	6647	0.08	102.5
GPX1	2876	0.16	82.5
SOD2	6648	0.28	33.1
GPX2	2877	0.44	0.33
TXNRD2	10587	0.49	1.00
GPX5	**2880**	**1.01**	**1.83**

^#^Signal-to-Noise Ratio (SNR): the differential expressed level against the background. The 1.5 × fold change and SNR≧1 as the criteria for selecting the significant differentially expressed genes (marked in bold).

## Data Availability

(1) The data used to support the findings of this study are included within the article. (2) Gene Expression Omnibus (GEO) Database: the GEO databases used in this study are the Lee dataset (GSE4536), Sun dataset (GSE4290), Murat dataset (GSE7696), and Shai dataset. These databases were used to assess gene expression levels in normal and glioma tissues.

## Supplementary Materials

Supplementary 1 Supplementary Figure 1: the body weight was measured every 7 days after xenograft with T98G stable clones. Bars represent the means ± SEM from three independent experiments. Differences among groups were determined with Nested one-way ANOVA followed by Dunnett's multiple comparison test. ns: no significant; shLuc: shRNA for luciferase; shB7, shC7: shRNAs for CEBPD.

Supplementary 2 Supplementary Figure 2: reduced CEBPD expression attenuates cell viability and induces cell apoptosis in GBM. (A) The U373MG or T98G cells were transiently transfected with control siRNA or CEBPD siRNA for 72 h and then subjected to CCK-8 viability assay. (B) Caspase 3/7 activity is increased in CEBPD knockdown GBM cells. Cells were harvested from U373MG or T98G stable clones, and caspase 3/7 activity was determined by. (C) The U373MG or T98G cells were transiently transfected with control siRNA or CEBPD siRNAs for 72 h and then stained with Annexin V and Propidium Iodide (PI) for flow cytometry analysis. Bars represent the means ± SEM from three independent experiments. Differences among groups were determined with one-way or two-way ANOVA followed by Tukey's multiple comparison test. ^∗∗∗^*p* < 0.001, ^∗∗^*p* < 0.01, and ^∗^*p* < 0.05. ns: no significant; shLuc: shRNA for luciferase; shB7, shC7: shRNAs for CEBPD; WT: parental cells; siNeg: siRNA for negative control; si2895, si2896: siRNAs for CEBPD.

Supplementary 3 Supplementary Figure 3: cell cycle analysis of control and CEBPD knockdown cells. Cells from stable clones (A) or transiently transfected (B) were fixed with ethanol and then stained with Propidium Iodide followed by fluorescence-activated cell sorter analysis. Bars represent the means ± SEMs from three independent experiments. Differences among groups were determined with one-way ANOVA followed by Tukey's multiple comparison test. ^∗∗∗^*p* < 0.001, ^∗∗^*p* < 0.01, and ^∗^*p* < 0.01. ns: no significant; shLuc: shRNA for luciferase; shB7, shC7: shRNAs for CEBPD; siNeg: siRNA for negative control; si2895, si2896: siRNAs for CEBPD.

Supplementary 4 Supplementary Figure 4: CEBPD activates CAT in GBM cells. (A) The U373MG or T98G cells were transiently transfected with control siRNA or CEBPD siRNA. After 72 h, total RNA and protein were harvested and examined by RT-qPCR (left panel) and Western blot analysis (right panel) with the indicated antibodies. (B) CEBPD activates CAT reporters in GBM cells. Left panel shows a schematic representation of the various CAT-based reporter constructs used in this study. Cells from U373MG or T98G stable clones were transfected with the indicated CAT reporter constructs. After 24 h, cells were lysed for luciferase assay. (C) The U373MG or T98G cells were cotransfected with siRNAs and HA or HA-CEBPD and CAT reporter construct (-1015~+49) and then examined by luciferase assay. (D) The U373MG or T98G cells were transiently transfected with control siRNA or CEBPD siRNA. After 72 h, cells were harvested and subjected to catalase activity analysis. Bars represent the means ± SEMs from three independent experiments. Differences among groups were determined with one-way or two-way ANOVA followed by Tukey's multiple comparison test. ^∗∗∗^*p* < 0.001, ^∗∗^*p* < 0.01, and ^∗^*p* < 0.05. siNeg: siRNA for negative control; si2895, si2896: siRNAs for CEBPD; HA: hemagglutinin; HA-CEBPD: HA-tagged CEBPD.
