# Supplementary material for: CCAAT/Enhancer-Binding Protein Delta Regulates Glioblastoma Survival through Catalase-Mediated Hydrogen Peroxide Clearance
Source: Oxid Med Cell Longev. 2022 Aug 18;2022:4081380. doi: 10.1155/2022/4081380 (PMC9411925; doi:10.1155/2022/4081380)
Supplement: Supplementary 1 — Supplementary Figure 1: the body weight was measured every 7 days after xenograft with T98G stable clones. Bars represent the means ± SEM from three independent experiments. Differences among groups were determined with Nested one-way ANOVA followed by Dunnett's multiple comparison test. ns: no significant; shLuc: shRNA for luciferase; shB7, shC7: shRNAs for CEBPD. [file 4081380.f1.pdf]

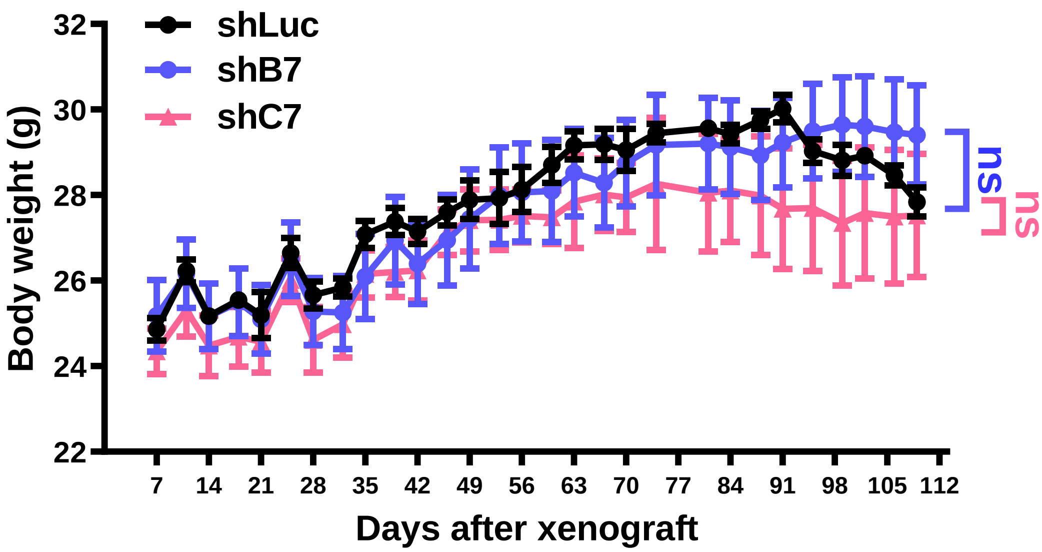

Supplementary figure 1. The body weight was measured every 7 days after xenograft with T98G stable clones. Bars represent the means ± SEM from three independent experiments. Differences among groups were determined with Nested one-way ANOVA followed by Dunnett's multiple comparison test. ns: no significant; shLuc: shRNA for Luciferase; shB7, shC7: shRNAs for CEBPD.
