# Supplementary material for: CCAAT/Enhancer-Binding Protein Delta Regulates Glioblastoma Survival through Catalase-Mediated Hydrogen Peroxide Clearance
Source: Oxid Med Cell Longev. 2022 Aug 18;2022:4081380. doi: 10.1155/2022/4081380 (PMC9411925; doi:10.1155/2022/4081380)
Supplement: Supplementary 2 — Supplementary Figure 2: reduced CEBPD expression attenuates cell viability and induces cell apoptosis in GBM. (A) The U373MG or T98G cells were transiently transfected with control siRNA or CEBPD siRNA for 72 h and then subjected to CCK-8 viability assay. (B) Caspase 3/7 activity is increased in CEBPD knockdown GBM cells. Cells were harvested from U373MG or T98G stable clones, and caspase 3/7 activity was determined by. (C) The U373MG or T98G cells were transiently transfected with control siRNA or CEBPD siRNAs for 72 h and then stained with Annexin V and Propidium Iodide (PI) for flow cytometry analysis. Bars represent the means ± SEM from three independent experiments. Differences among groups were determined with one-way or two-way ANOVA followed by Tukey's multiple comparison test. ∗∗∗p < 0.001, ∗∗p < 0.01, and ∗p < 0.05. ns: no significant; shLuc: shRNA for luciferase; shB7, shC7: shRNAs for CEBPD; WT: parental cells; siNeg: siRNA for negative control; si2895, si2896: siRNAs for CEBPD. [file 4081380.f2.pdf]

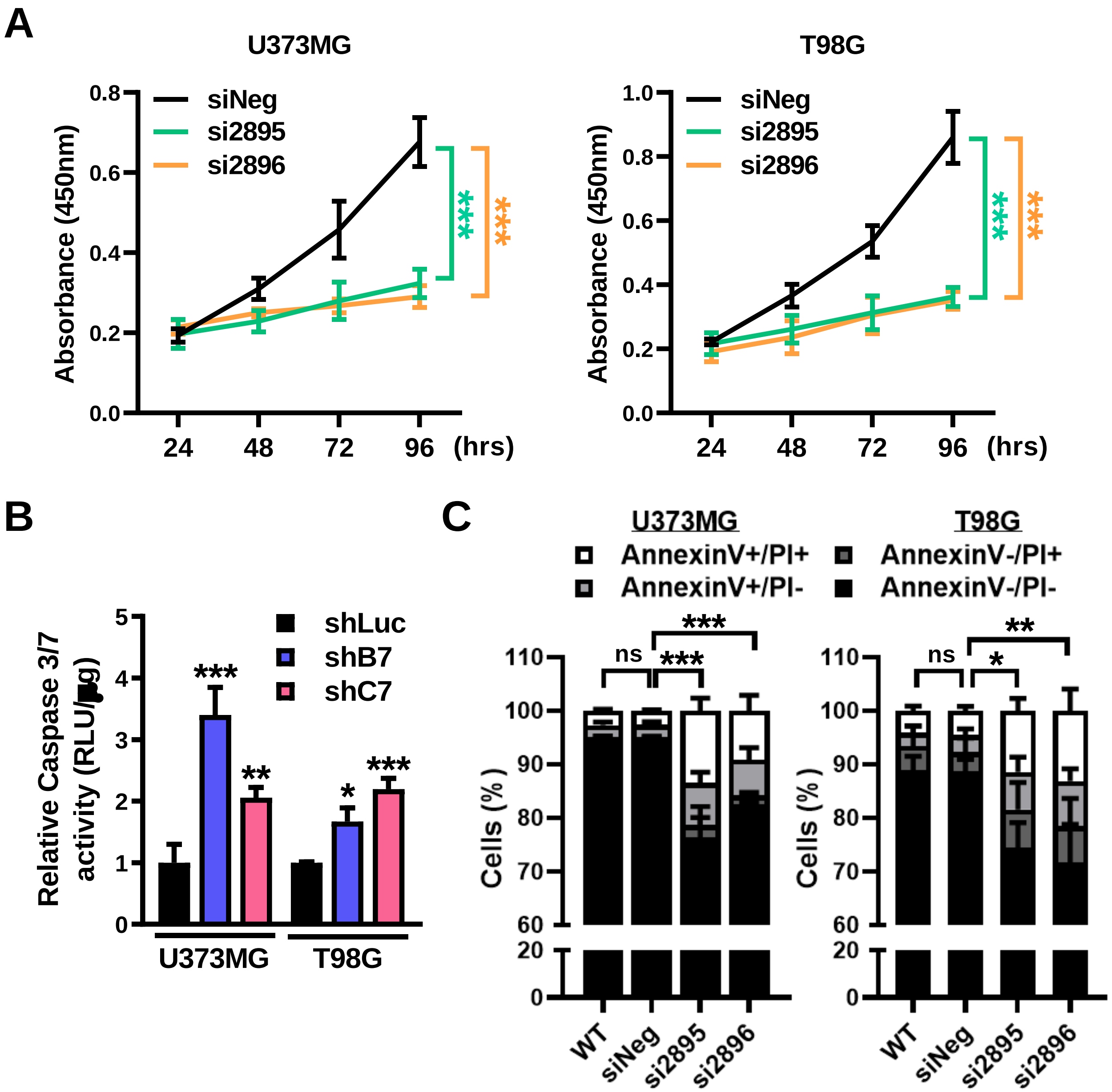

Supplementary figure 2. Reduced CEBPD expression attenuates cell viability and induces cell apoptosis in GBM. (A) The U373MG or T98G cells were transiently transfected with control siRNA or CEBPD siRNA for 72 h and then subjected to CCK-8 viability assay. (B) Caspase 3/7 activity is increased in CEBPD knockdown GBM cells. Cells were harvested from U373MG or T98G stable clones and caspase-3/7 activity was determined by Caspase-Glo® 3/7 Reagent. (C) The U373MG or T98G cells were transiently transfected with control siRNA or CEBPD siRNAs for 72 h and then stained with Annexin V and Propidium Iodide (PI) for flow cytometry analysis. Bars represent the means ± SEM from three independent experiments. Differences among groups were determined with one-way or two-way ANOVA followed by Tukey's multiple comparison test. ***$p < 0.001$, **$p < 0.01$, *$p < 0.05$. ns: no significant; shLuc: shRNA for Luciferase; shB7, shC7: shRNAs for CEBPD; WT: parental cells; siNeg: siRNA for Negative control; si2895, si2896: siRNAs for CEBPD.
