# Supplementary material for: CCAAT/Enhancer-Binding Protein Delta Regulates Glioblastoma Survival through Catalase-Mediated Hydrogen Peroxide Clearance
Source: Oxid Med Cell Longev. 2022 Aug 18;2022:4081380. doi: 10.1155/2022/4081380 (PMC9411925; doi:10.1155/2022/4081380)
Supplement: Supplementary 3 — Supplementary Figure 3: cell cycle analysis of control and CEBPD knockdown cells. Cells from stable clones (A) or transiently transfected (B) were fixed with ethanol and then stained with Propidium Iodide followed by fluorescence-activated cell sorter analysis. Bars represent the means ± SEMs from three independent experiments. Differences among groups were determined with one-way ANOVA followed by Tukey's multiple comparison test. ∗∗∗p < 0.001, ∗∗p < 0.01, and ∗p < 0.01. ns: no significant; shLuc: shRNA for luciferase; shB7, shC7: shRNAs for CEBPD; siNeg: siRNA for negative control; si2895, si2896: siRNAs for CEBPD. [file 4081380.f3.pdf]

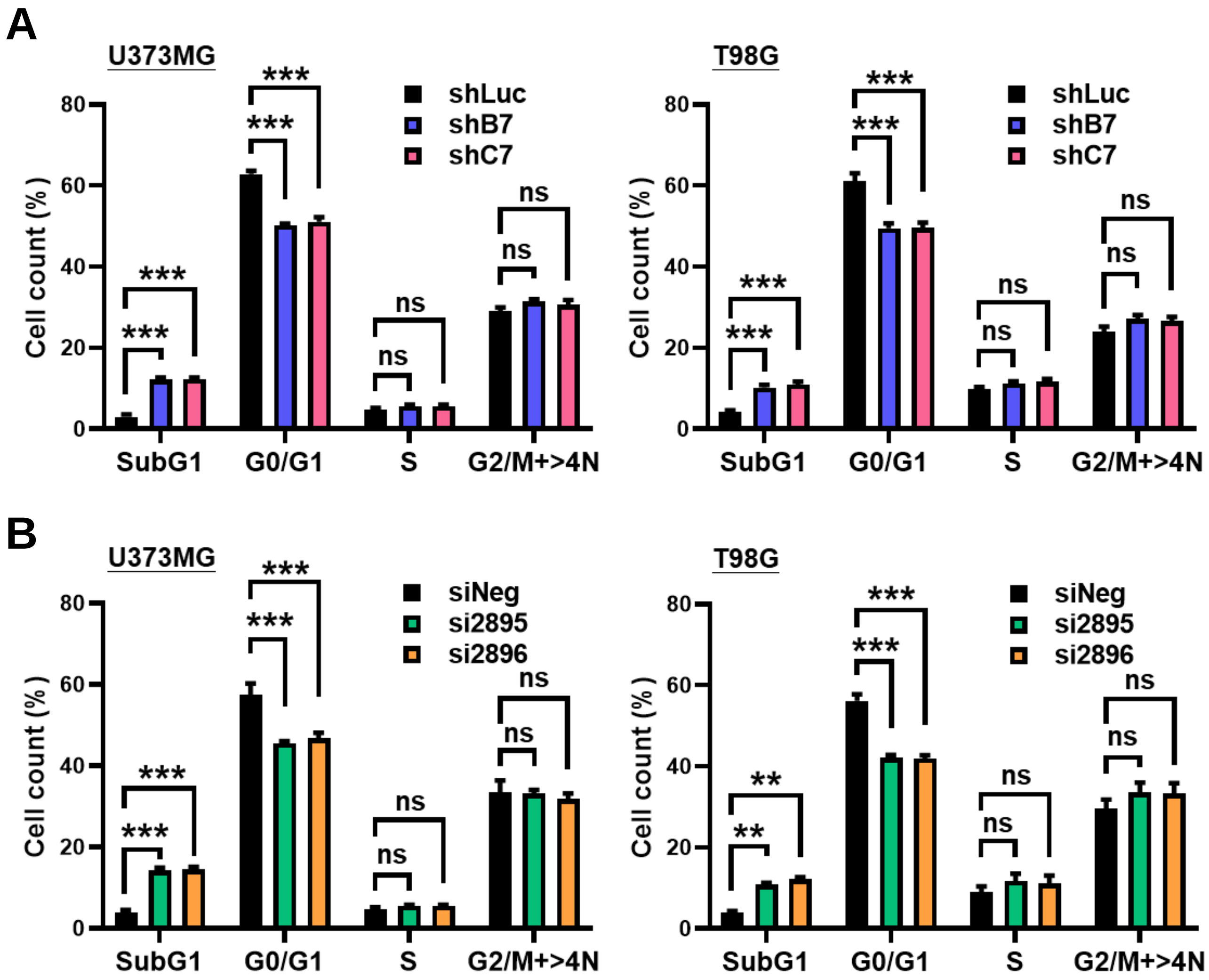

Supplementary figure 3. Cell cycle analysis of control and CEBPD knockdown cells. Cells from stable clones (A) or transiently transfected (B) were fixed with ethanol and then stained with propidium iodide followed by fluorescence-activated cell sorter analysis. Bars represent the means ± SEMs from three independent experiments. Differences among groups were determined with one-way ANOVA followed by Tukey's multiple comparison test. ***$p < 0.001$, **$p < 0.01$, *$p < 0.01$. ns: no significant; shLuc: shRNA for Luciferase; shB7, shC7: shRNAs for CEBPD; siNeg: siRNA for Negative control; si2895, si2896: siRNAs for CEBPD.
