# Supplementary material for: CCAAT/Enhancer-Binding Protein Delta Regulates Glioblastoma Survival through Catalase-Mediated Hydrogen Peroxide Clearance
Source: Oxid Med Cell Longev. 2022 Aug 18;2022:4081380. doi: 10.1155/2022/4081380 (PMC9411925; doi:10.1155/2022/4081380)
Supplement: Supplementary 4 — Supplementary Figure 4: CEBPD activates CAT in GBM cells. (A) The U373MG or T98G cells were transiently transfected with control siRNA or CEBPD siRNA. After 72 h, total RNA and protein were harvested and examined by RT-qPCR (left panel) and Western blot analysis (right panel) with the indicated antibodies. (B) CEBPD activates CAT reporters in GBM cells. Left panel shows a schematic representation of the various CAT-based reporter constructs used in this study. Cells from U373MG or T98G stable clones were transfected with the indicated CAT reporter constructs. After 24 h, cells were lysed for luciferase assay. (C) The U373MG or T98G cells were cotransfected with siRNAs and HA or HA-CEBPD and CAT reporter construct (-1015~+49) and then examined by luciferase assay. (D) The U373MG or T98G cells were transiently transfected with control siRNA or CEBPD siRNA. After 72 h, cells were harvested and subjected to catalase activity analysis. Bars represent the means ± SEMs from three independent experiments. Differences among groups were determined with one-way or two-way ANOVA followed by Tukey's multiple comparison test. ∗∗∗p < 0.001, ∗∗p < 0.01, and ∗p < 0.05. siNeg: siRNA for negative control; si2895, si2896: siRNAs for CEBPD; HA: hemagglutinin; HA-CEBPD: HA-tagged CEBPD. [file 4081380.f4.pdf]

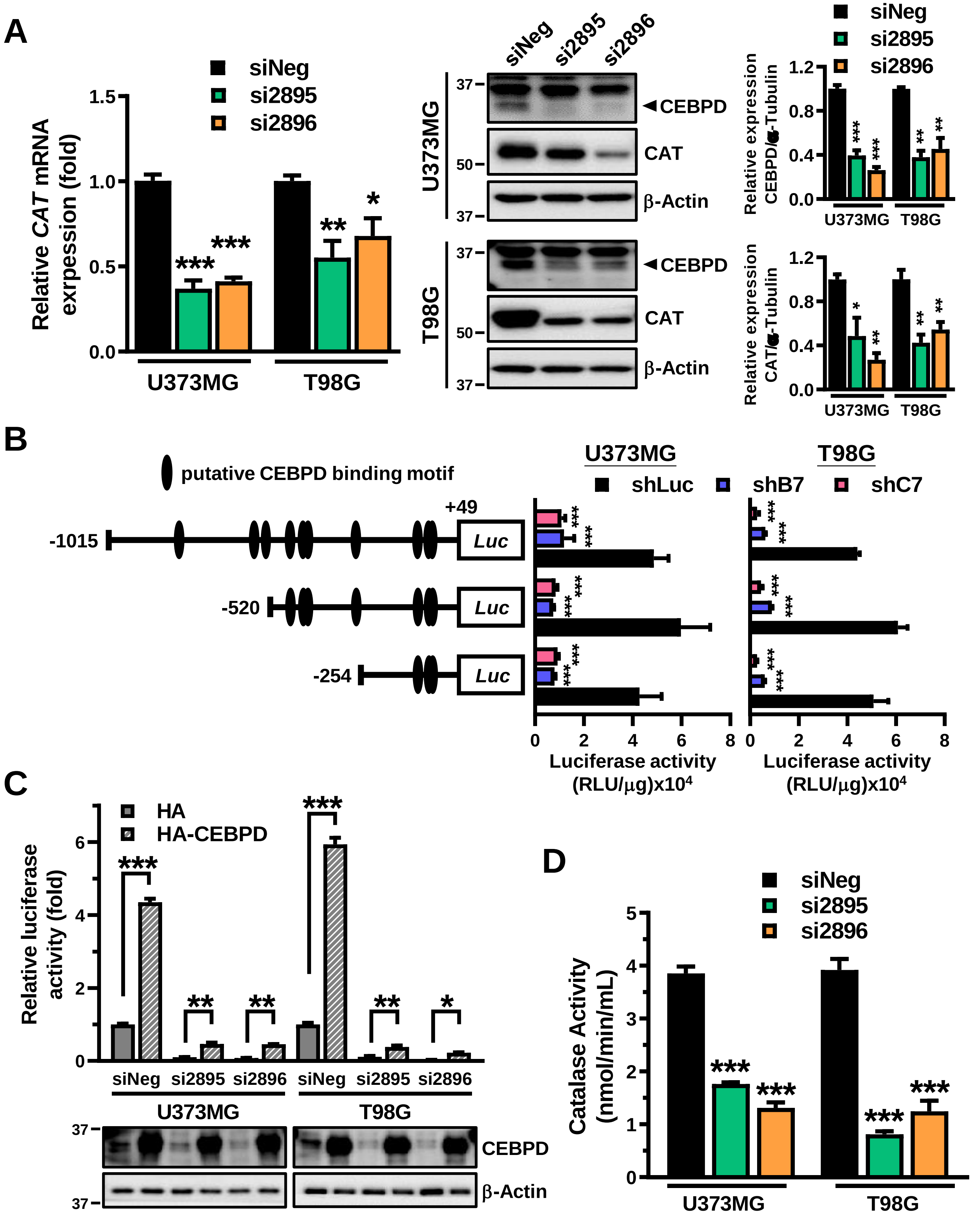

Supplementary figure 4.

Supplementary figure 4. CEBPD activates CAT in GBM cells. (A) The U373MG or T98G cells were transiently transfected with control siRNA or CEBPD siRNA. After 72 h, total RNA and protein were harvested and examined by RT-qPCR (left panel) and Western blot analysis (right panel) with the indicated antibodies. (B) CEBPD activates CAT reporters in GBM cells. Left panel shows a schematic representation of the various CAT-based reporter constructs used in this study. Cells from U373MG or T98G stable clones were transfected with the indicated CAT reporter constructs. After 24 h, cells were lysed for luciferase assay. (C) The U373MG or T98G cells were co-transfected with siRNAs and HA or HA-CEBPD and CAT reporter construct (-1015~+49) and then examined by luciferase assay. (D) The U373MG or T98G cells were transiently transfected with control siRNA or CEBPD siRNA. After 72 h, cells were harvested and subjected to catalase activity analysis. Bars represent the means ± SEMs from three independent experiments. Differences among groups were determined with one-way or two-way ANOVA followed by Tukey's multiple comparison test. ***$p < 0.001$, **$p < 0.01$, *$p < 0.05$. siNeg: siRNA for Negative control; si2895, si2896: siRNAs for CEBPD; HA: hemagglutinin; HA-CEBPD: HA-tagged CEBPD.
